# Lipin-1 Deficiency-Associated Recurrent Rhabdomyolysis and Exercise-Induced Myalgia Persisting into Adulthood: A Case Report and Review of Literature

**DOI:** 10.1155/2020/7904190

**Published:** 2020-05-27

**Authors:** Neluwa Liyanage Ruwan Indika, Dinesha Maduri Vidanapathirana, Eresha Jasinge, Roshitha Waduge, Narangoda Liyanage Ajantha Shyamali, Poruthotage Pradeep Rasika Perera

**Affiliations:** ^1^Department of Biochemistry, Faculty of Medical Sciences, University of Sri Jayewardenepura, Nugegoda, Sri Lanka; ^2^Department of Pathology, Faculty of Medical Sciences, University of Sri Jayewardenepura, Nugegoda, Sri Lanka; ^3^Department of Chemical Pathology, Lady Ridgeway Hospital for Children, Colombo 8, Sri Lanka; ^4^Department of Pathology, Faculty of Medicine, University of Peradeniya, Peradeniya, Sri Lanka; ^5^Department of Medicine, Faculty of Medical Sciences, University of Sri Jayewardenepura, Nugegoda, Sri Lanka

## Abstract

Phosphatidate phosphatase-1 (lipin-1) is encoded by *LPIN1* gene. Lipin-1 deficiency has been reported as the second most common cause of early-onset rhabdomyolysis after primary fatty acid oxidation disorders. We report a case of a 32-year-old Sri Lankan female with a history of more than 10 episodes of rhabdomyolysis and exercise intolerance since childhood. These episodes were triggered by infections and exercise. A temporal relationship between the acute episodes and use of drugs such as theophylline, mefenamic acid, co-trimoxazole, and combined oral contraceptive pills was also noted. There was marked elevation of serum creatine kinase and transaminases during acute episodes. Family history revealed parental consanguinity and an affected sibling who died of an acute episode associated with muscle weakness, dark coloured urine, and cyanosis, at the age of 2 years. The histochemical findings of the patient under discussion were consistent with a metabolic myopathy affecting membrane integrity. A homozygous, likely pathogenic variant c.1684G>*T* encoding p.(Glu562∗) was identified by clinical exome sequencing. Even though the studies to date give no convincing evidence of a possible causal or contributory relationship between the drugs under discussion and lipin-1 related rhabdomyolysis, this case highlights the importance of pharmacovigilance and reporting adverse drug reactions in patients with lipin-1 deficiency.

## 1. Introduction

Rhabdomyolysis (RML) is a serious and potentially life-threatening condition characterized by elevation of serum creatine kinase (sCK) activity to at least five times the upper limit of normal followed by a rapid decrease. Classical clinical features are subacute-onset myalgia, transient muscle weakness, and pigmenturia caused by an excessive amount of myoglobin in the urine [1]. A number of genetic disorders including glycogen storage disorders, disorders of fatty acid oxidation, mitochondrial disorders, and phosphatidate phosphatase-1 (lipin-1) deficiency are associated with episodes of RML [2].

Lipin-1 is a magnesium-dependant enzyme encoded by *LPIN1* gene (OMIM #605518) having chromosomal location *2p25.1* [3]. Mutations in this gene are associated with acute recurrent RML and exercise-induced myalgia. Episodes of RML are mostly precipitated by febrile illnesses and occasionally by intense exercise, anesthesia, and/or fasting [4, 5]. Lipin-1 deficiency is an autosomal recessive genetic disorder which belongs to a new group of inherited metabolic disorders classified as defects of complex lipid biosynthesis and remodeling [6, 7].

## 2. Case Report

We report a case of a 32-year-old female who presented with a history of recurrent myalgia and weakness associated with dark coloured urine since childhood. At the age of 1 year, she has had her first acute episode which was associated with fever and sudden onset weakness of all four limbs. On admission, she was cyanosed, the respiratory rate was 60/min, and the heart rate was 120/min which increased to 180/min on the second day. Neurological examination revealed muscle power of grade 2 (movement possible if gravity eliminated), hypotonia with diminished reflexes. Serial electrocardiograms showed sinus tachycardia. She was able to stand up with support after two weeks of admission. This episode was diagnosed as a “viral myocarditis with polyneuritis” and managed with a course of prednisolone for 19 days.

The second and third acute episodes that occurred at the age of 18 and 20 months, respectively, were diagnosed as “familial periodic paralysis.” Fourth episode at the age of 22 months was associated with fever, muscle weakness, passage of red coloured urine, and difficulty in breathing. This has been attributed to a questionable hemoglobinuria precipitated by co-trimoxazole given for a suspected infection, and therefore, the child was investigated for glucose-6-phosphate dehydrogenase deficiency. Familial paroxysmal myoglobinuria was first suspected on her fifth episode at the age of 4  years with the detection of oxymyoglobin and metmyoglobin in urine. Watson and Schwartz test for porphobilinogen performed during the 5^th^ acute episode was negative. Nevertheless, the diagnostic card of the sixth episode (at 4 and half years) mentioned a questionable diagnosis of an acute intermittent porphyria (AIP). Subsequent acute episodes which occurred at the ages of 6, 9, 22, 28, and 30 years were of varying severity. Some of these episodes were claimed to be triggered by theophylline given for a wheezing attack, mefenamic acid given for a toothache, and starting doses of combined oral contraceptive pills taken for family planning. Severe muscle pain and muscle weakness resolved soon after withholding the drugs. She is on long-term inhaled steroids and bronchodilators (salbutamol, salmeterol, and fluticasone) for bronchial asthma which were not associated with any adverse events.

She has been investigated for primary subfertility of two years. A laparoscopic dye test has been postponed due to questionable diagnosis of an AIP which would have implications in anesthesia. She is living with her husband who is employed in an overseas country. She sought our assistance in order to get a definitive diagnosis for her condition when she visited Sri Lanka.

In-between episodes, the patient experiences exercise intolerance. She describes that during exertion, she feels a numbness and coldness starting from the calf spreading up to the thighs and then affecting the upper limbs. As she had to continue exertion beyond this point for exercise ECG test, the fatigue progressed to severe shortness of breath associated with nausea and vomiting.

**Figure 1:**
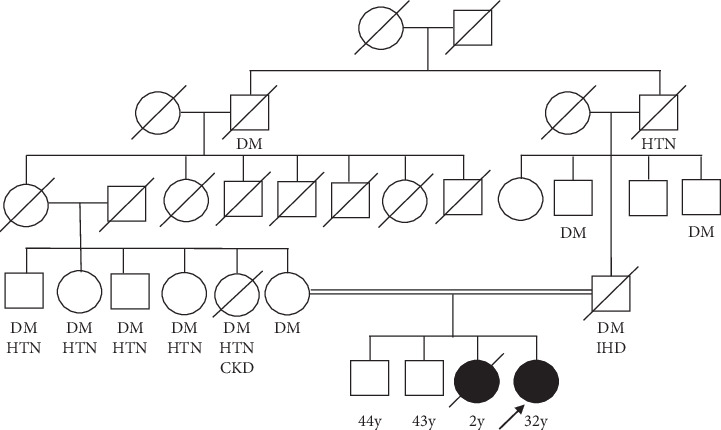
Pedigree of the index patient showing a strong family history of diabetes mellitus and hypertension. DM, diabetes mellitus; HTN, hypertension; IHD, ischaemic heart disease; CKD, chronic kidney disease.

Her parents are consanguineous. There is a strong family history of diabetes mellitus and hypertension (Figure 1). Her sister has suffered from similar clinical features and died of a similar acute episode associated with muscle weakness, dark coloured urine, and cyanosis, at the age of 2 years.

The primary examination reported here was conducted 2 years after the last episode of RML and when the patient was asymptomatic. On examination, her body mass index (BMI) was 25.7  kg/m^2^ (normal; 18.5–22.9). Resting pulse rate was 110/min. Systemic blood pressure was 110/60  mmHg. Precordial examination was clinically normal. Upper limb and lower limb muscle power was grade 4 (movements possible against a moderate resistance). Deep tendon reflexes were normal.

**Figure 2:**
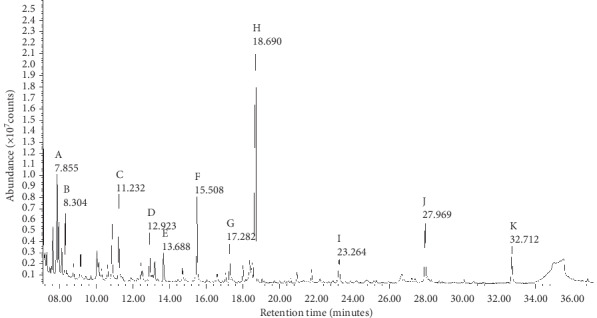
Gas chromatography–mass spectrometry (GC-MS) total ion chromatogram of urine organic acids when the patient was asymptomatic. Letters indicate the following metabolites: (a) lactate; (b) glycolate; (c) 3-hydroxyisobutyrate; (d) 3-hydroxyisovalerate; (e) 2-ethylhydracrylate; (f) ethylmalonate; (g) unknown; (h) internal standard; (i) 4-hydroxycyclohexanecarboxylate; (j) 4-hydroxylphenylacetate; (k) unknown.

Results of biochemical investigations done during the past episodes are summarized in Table 1. Among the abnormalities observed were marked elevation of sCK and transaminases. Urine organic acid profile was normal (Figure 2). Plasma amino acids were analyzed using high-performance liquid chromatography. The amino acid profile revealed nonspecific reduction of some amino acids (Table 2).

Ultrasound examination of the abdomen revealed a grade 1 fatty liver. Body composition analysis using a bioimpedance scale revealed normal muscle mass 34.9% (33.2–37.8%) with increased fat percentage 40.2% (21–35%). Visceral fat rating was normal (<10%). The total muscle mass was average with lesser rating for truncal muscle mass compared to limb muscles on segmental analysis of muscle mass (supplementary material).

**Figure 3:**
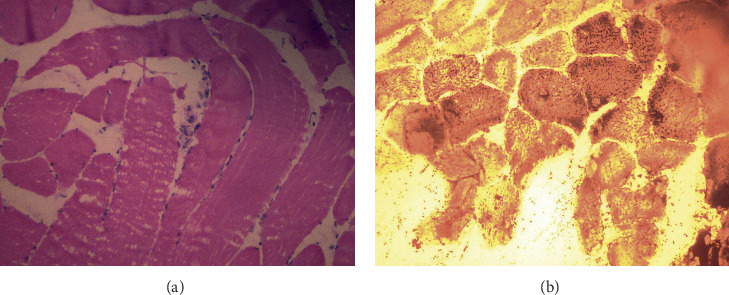
(a) H&E sections of frozen muscle showing skeletal muscle fibers with occasional myofiber necrosis and disruption of sarcolemma by inflammatory cell infiltrates (200x magnification). (b) Sudan black stains of the muscle biopsy showing occasional subsarcolemmal aggregates of lipid droplets (200x magnification).

A muscle biopsy was taken two years after the last episode of RML, when the patient was asymptomatic. Haematoxylin and eosin (H&E) staining of the tissue sections showed skeletal muscle fibers with occasional myofiber necrosis and disruption of sarcolemma by inflammatory cell infiltrate. Focal fiber atrophy was identified (Figure 3(a)). Ragged red fibers or vacuolation was not seen with Gomori trichrome and Periodic acid–Schiff (PAS) stains, respectively. Oil Red O stain revealed minute lipid droplets in some fibers but large droplet accumulation was not seen (images not shown). Sudan black stain revealed occasional subsarcolemmal aggregates of lipid droplets in a few fibers (Figure 3(b)). The findings are consistent with a metabolic myopathy affecting the membrane integrity.

Clinical exome sequencing was carried out in an overseas laboratory. Next generation sequencing was performed. Genomic DNA was enzymatically fragmented and regions of interest were selectively enriched using capture probes targeted against coding regions of ∼6700 genes with known clinical significance. Libraries were generated with Illumina compatible adaptors and sequenced on an Illumina platform. Evaluation was focused on coding exons along with flanking ± 10 intronic bases within the captured region. A homozygous likely pathogenic variant c.1684 G > *T* encoding p.(Glu562∗) was identified in the *LPIN1* gene (NM_001261428.2).

The patient was given advice in order to avoid trigger factors such as fasting for more than 8 hours, excessive or prolonged exertion, and probable precipitants (theophylline, co-trimoxazole, and mefenamic acid). Need for immediate medical consultation in case of muscle pain, muscle weakness, and fever was emphasized. An informative diagnostic card was provided with recommendations pertaining to management of acute episodes, preoperative care, anaesthesia, and pharmacotherapy.

Two weeks after arriving at a definitive diagnosis, the patient migrated overseas again and had to engage in extensive physical activities without adequate rest, which had precipitated an acute episode. She sought medical advice with the diagnostic card and the recommendations. She was admitted to an emergency care unit and hydrated with 0.9% normal saline. The patient was given a high calorie diet with extra carbohydrates included in main meals, snacks, desserts, and beverages. Severity of RML was monitored with sCK levels and urine myoglobin. The patient was discharged after three days as the symptoms settled and sCK levels decreased.

## 3. Discussion

*LPIN1-*related RML manifests usually in children less than 6 years and occasionally in adults. In children, the episodes of RML are severe enough to cause death [8, 9]. The first attack of RML was the most severe acute episode of our patient as well. Decreasing frequency and intensity of episodes with advancing age is consistent with the clinical course described in previous studies. Severe attacks are associated with life-threatening cardiac arrhythmias as observed in this patient and previously reported cases [4]. Lipin-1 is highly expressed in myocardium and involved in fatty acid metabolism in the cardiomyocytes [10]. Therefore, lipin-1 deficiency itself can potentially have deleterious effects on cardiac functions. Hyperkalemia and arrhythmogenic effects of orally administered bronchodilators may also contribute to development of cardiac arrhythmias during acute episodes. These cases highlight the importance of cardiac monitoring and cautious use of medicines in lipin-1 deficiency.

In a case series of six individuals who developed statin-induced myopathy, two were carriers for heterozygous mutations in the *LPIN1* gene [11]. There are a number of case reports of RML following administration of theophylline [12, 13]. A few case reports have reported co-trimoxazole as a potential trigger of RML [14, 15]. Even though endogenous estrogen appears to attenuate muscle damage in animals, women taking oral contraceptives, thereby having higher exogenous estrogen levels with lower endogenous estrogen levels, appear to be more susceptible to exercise-induced muscle damage and have an attenuated recovery from exercise-induced muscle damage [16–18]. There are a few case reports of RML developing after administration of diclofenac sodium, but RML following administration of mefenamic acid has not been reported [19–21]. Nonsteroidal anti-inflammatory drugs (NSAIDs), such as diclofenac sodium and mefenamic acid, have been suggested to cause cell injury by inducing proteasome dysfunction and uncoupling mitochondrial oxidative phosphorylation, resulting in decreased ATP synthesis [22, 23]. Respiratory tract infections together with pharmacological agents such as theophylline, steroids, and beta adrenergic receptor agonists may cooperate together in an acute exacerbation of bronchial asthma to unmask underlying metabolic myopathies such as carnitine palmitoyltransferase deficiency and lipin-1 deficiency [24, 25]. However, the presumptive link between the drugs and RML in lipin-1 deficiency as observed in this patient is poorly supported by published work to date. Therefore, case reports should explore possible new adverse drug reactions in patients with this disorder and form the basis for causality assessment in future studies.

Lipin-1-deficient (*fld*) mice exhibit adipose tissue deficiency and reduced muscle mass [26, 27]. Nevertheless, deleterious mutations of human *LPIN1* do not affect adipose tissue distribution. It has been suggested that induction of alternative pathways for triacylglycerol synthesis takes place in human adipocytes under conditions of repressed lipin expression [28]. It is also suggested that lipin-2 expression in adipose tissue may compensate for lack of lipin-1, as lipin-2 expression has been detected in human adipose tissue [29]. The subject under discussion is overweight and had high total fat percentage with average visceral fat rating. The segmental analysis of muscle mass of the limbs were average with diminished muscle power. However, in a previous case study, physical examination of patients aged between 8 and 10  years showed normal fat distribution and muscle strength [11]. Further studies are needed to determine pattern of the muscle distribution and muscle strength in patients with lipin-1 deficiency.

Both maternal and paternal branches of the pedigree consist of a significant number of individuals affected with metabolic disorders such as diabetes mellitus and hypertension. Lipin-1 expression in humans is positively correlated with insulin sensitivity, and expression of genes is involved in lipid oxidation, uptake, and lipolysis [30–32]. It is questionable if the heterozygous status has contributed to these unfavorable phenotypic features in the pedigree. Mutations in *LPIN1* gene cause hepatic steatosis, reduce lipoprotein lipase activity, and hepatic lipase activity in *fld* mice [33]. Lipin-1 deficiency may be implicated in development of fatty liver of the patient.

RML leads to release of intracellular enzymes such as creatine kinase, aminotransferases, and lactate dehydrogenase into the systemic circulation. An increase of sCK to a level more than five times the upper limit of normal in the absence of myocardial or cerebral infarction indicates significant muscle damage. Leukocytosis may also accompany RML. Myoglobinuria is not always visible and resolve early in the course of RML rendering this parameter less sensitive [34, 35]. In patients with suspected metabolic myopathies performing muscle biopsy and specific stains is helpful in narrowing down differential diagnosis [36]. Among the histological findings in skeletal muscles of lipin-1 deficiency are muscle cell necrosis, increase of quantity and size of lipid droplets in the muscle fibers, predominance of type I muscle fibers, and atrophy of type II fibers [2, 4, 9, 37].

More than twenty *LPIN1* mutations have been described to date in several ethnic groups [4, 11, 37–40]. The *LPIN1* variant c.1684 G > *T* encoding p. Glu562∗ also generates a premature stop codon resulting in synthesis of a truncated protein with loss of enzymatic activity. This variant has previously been reported as p. Glu477 ∗ due to use of a different reference sequence; NM_145693.1 [4]. It is classified as likely pathogenic (class 2) according to the recommendations of American College of Medical Genetics (ACMG). The spectrum of mutations is heterogeneous. Therefore, a gene sequencing panel or whole exome sequencing is required to confirm the diagnosis of this condition.

Once diagnosis is made, genetic counseling should be offered. Patient should be provided with a diagnostic card outlining the emergency management to facilitate early recognition of acute episodes and implementation of early monitoring and treatment of RML and related complications. During an acute episode, possible causative agent should be eliminated and infections should be treated with empirical antibiotics. Regular cardiac monitoring is important due to risk of developing life-threatening arrhythmias. Pichler et al. proposed that hyperhydration using 10% dextrose solutions at the first symptom of RML and administration of insulin to control hyperglycaemia and hyperkalaemia may reduce the duration of acute episodes of RML. In order to prevent future episodes of RML, trigger factors such as prolonged fasting and vigorous or prologed physical activities should be avoided. Furthermore, maintaining high caloric intake with oral carbohydrates in situations possibly leading to catabolism such as infections or excessive physical activities may reduce the frequency of episodes of RML. [41, 42]. Some case reports have proposed that use of fat-restricted diet supplemented with medium chain triglycerides and L-carnitine could be clinically effective [8].

## 4. Conclusion

In some instances, RML may be due to a combination of underlying genetic disorder and environmental triggers. Identifying underlying genetic disorders presenting with RML can pose a diagnostic challenge due to their rarity, marked heterogeneity, and nonspecific clinical features requiring a high index of suspicion. Recurrent RML, positive family history, concomitant presence of exercise intolerance, or recurrent muscle cramps should raise a suspicion of lipin-1 deficiency after exclusion of primary fatty acid oxidation disorders. Lipin-1 deficiency associated RML may manifest in adults. The case report also highlights the importance of pharmacovigilance and reporting any adverse drug reactions in patients with lipin-1 deficiency taking into consideration the possible link between drugs and lipin-1 related RML.

## Figures and Tables

**Table 1:** General biochemical investigations done during acute episodes and when symptom free.

Test	5th attack	7th attack	8th attack	Baseline	Unit
Serum Na^+^	124 (135–145)	130.2 (135–145)	139.3 (135–145)	139 (130–145)	mmol/L
Serum K^+^	5.2 (3.5–5.3)	4.5 (3.5–5.3)	4.3 (3.5–5.3)	4.2 (3.5–5.3)	mmol/L
White blood cell count	25.7 (4–11)	15.9 (4–11)	Not recorded	13.9 (4–11)	×10^3^/*μL*
Neutrophil%	91% (40–60%)	81% (40–60%)	Not recorded	80.1% (40–60%)	%
Lymphocyte%	9% (20–40%)	19% (20–40%)	Not recorded	12.9% (20–40%)	%
sCK	>20000 (34–145)	Not recorded	>5000 (34–145)	224 (34–145)	U/L
Serum CK(MB)	Not recorded	Not recorded	273 (<25)	Not done	U/L
Serum LDH	14850 (180–360)	Not recorded	Not recorded	Not done	U/L
Serum SGOT	>1000 (0–40)	415 (0–40)	Not recorded	25 (0–40)	U/L
Serum SGPT	>1000 (9–48)	77 (9–48)	Not recorded	82 (9–48)	U/L
Serum bilirubin (total)	10.26 (3–20)	5.13 (3–20)	Not recorded	15 (3–20)	*μ*mol/L

Reference intervals are given in parenthesis.

**Table 2:** Plasma amino acid profile of the patient.

Amino acid	Plasma amino acid concentration (*μ*mol/L)	Reference interval (*μ*mol/L)
Alanine	**193**	218–474
Arginine	39	28–108
Citrulline	20	10–58
Cystine	**13**	31–49
Glutamic acid	17	6–38
Glutamine	**263**	340–696
Glycine	**82**	100–384
Histidine	**33**	68–104
Isoleucine	**18**	39–67
Leucine	**50**	98–142
Lysine	**65**	119–203
Methionine	**10**	14–30
Ornithine	**20**	36–96
Phenylalanine	**25**	42–62
Serine	**33**	78–166
Taurine	21	18–66
Threonine	**39**	93–197
Tryptophan	24	17–53
Tyrosine	**19**	26–78
Valine	**96**	172–248

Abnormal values are in boldface.

## Supplementary Materials

Supplementary Materials Body composition of the patient measured by bioelectrical impedance analysis (BIA) technology (Tanita MC-980 Body Composition Analyzer).
