# Supplementary material for: Lipin-1 Deficiency-Associated Recurrent Rhabdomyolysis and Exercise-Induced Myalgia Persisting into Adulthood: A Case Report and Review of Literature
Source: Case Rep Med. 2020 May 27;2020:7904190. doi: 10.1155/2020/7904190 (PMC7275236; doi:10.1155/2020/7904190)

Body composition of the patient measured by bioelectrical impedance analysis (BIA) technology

(Tanita MC-980 Body Composition Analyzer)

Details

MC-780	Result	Desirable	Target	
Weight	61.8 kg	44.4-60.1 kg	kg	kg
Fat	40.2 %	21.0-35.0 %	%	%
Fat Mass	24.8 kg	9.8-19.9 kg	kg	kg
FFM	37.0 kg			
Muscle Mass	34.9 kg	33.2-37.8		
BMI	25.7	18.5-25.0		
Metabolic Age	48.0			

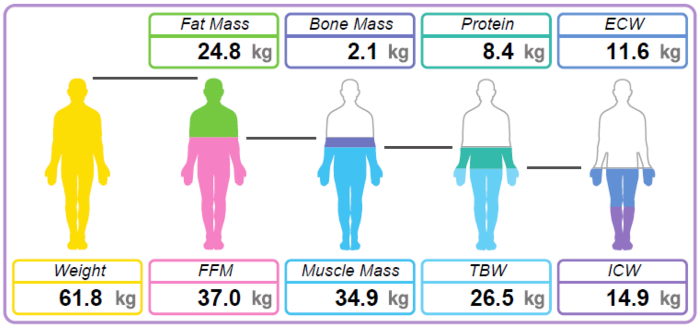

BMR VFA TBW

BMR 4827 kJ / 1153 kcal (Under | Normal | More)

Visceral Fat Rating 7 (10, 15; Average | High | Very High)

TBW 26.5 kg / 42.9 % — **ECW** 11.6 kg — **ICW** 14.9 kg

ECW/TBW 43.8 % (35% | 40% | 45%)

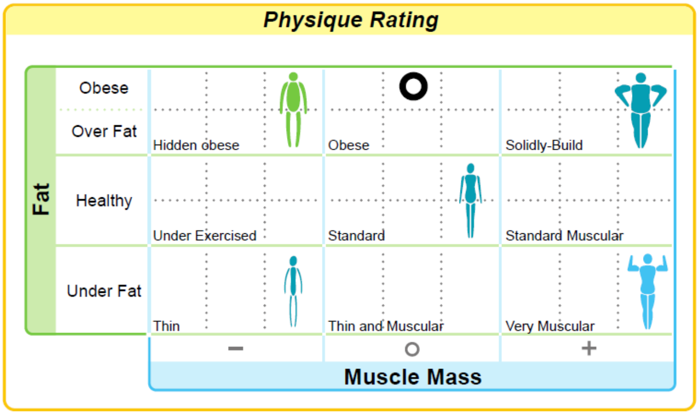

Segmental Analysis

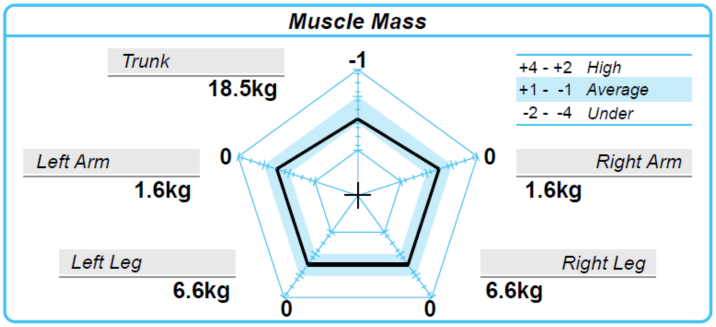

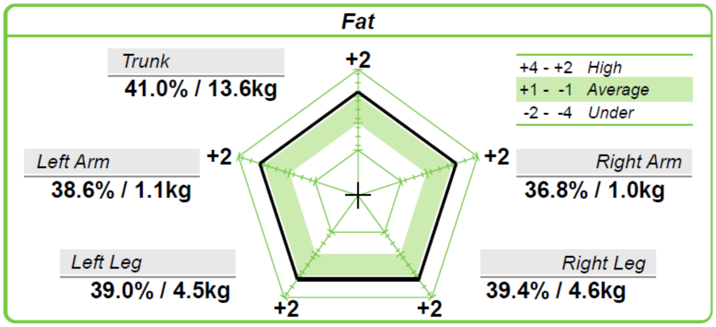

Balance

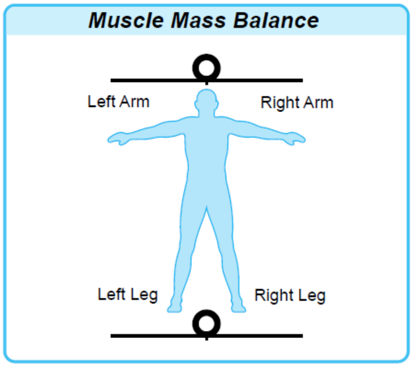

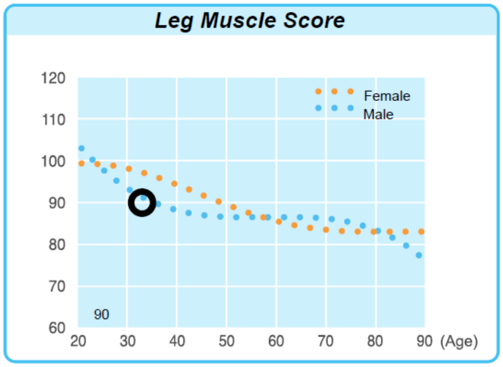

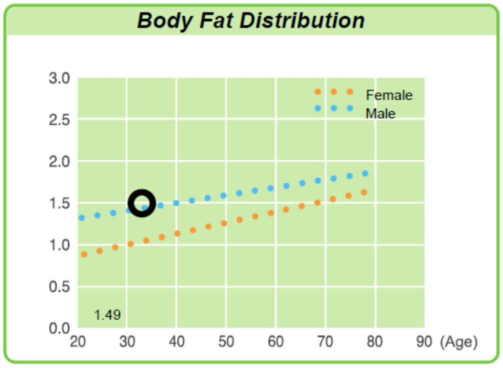

Supplement: Supplementary Materials — Body composition of the patient measured by bioelectrical impedance analysis (BIA) technology (Tanita MC-980 Body Composition Analyzer). [file 7904190.f1.pdf]
